# Developing a risk prediction tool for lung cancer in Kent and Medway, England: cohort study using linked data

**DOI:** 10.1038/s44276-023-00019-5

**Published:** 2023-10-17

**Authors:** David Howell, Ross Buttery, Padmanabhan Badrinath, Abraham George, Rithvik Hariprasad, Ian Vousden, Tina George, Cathy Finnis

**Affiliations:** 1 Quantum Analytica, Berkshire, UK; 2 Surrey Heartlands Integrated Care System, Surrey, UK; 3 Public Health Medicine, Kent County Council, Maidstone, England UK; 4 University of Cambridge, Cambridge, UK; 5 Kent and Medway Medical School, Kent, UK; 6 Vellore Institute of Technology, Vellore, Tamil Nadu India; 7 Thames Valley Cancer Alliance, Reading, UK; 8 NHS England - South East, Southampton, UK; 9 Kent & Medway Cancer Alliance, Maidstone, UK; 10 Targeted Lung Health Checks, Sussex, UK; 11 NHS Sussex Integrated Care Board, Worthing, England UK; 12 Cancer Research UK GP, London, UK; 13 Early Cancer Diagnosis and Cancer Health Inequalities, Kent and Medway Cancer Alliance, Maidstone, UK

## Abstract

**Background:**

Lung cancer has the poorest survival due to late diagnosis and there is no universal screening. Hence, early detection is crucial. Our objective was to develop a lung cancer risk prediction tool at a population level.

**Methods:**

We used a large place-based linked data set from a local health system in southeast England which contained extensive information covering demographic, socioeconomic, lifestyle, health, and care service utilisation. We exploited the power of Machine Learning to derive risk scores using linear regression modelling. Tens of thousands of model runs were undertaken to identify attributes which predicted the risk of lung cancer.

**Results:**

Initially, 16 attributes were identified. A final combination of seven attributes was chosen based on the number of cancers detected which formed the Kent & Medway lung cancer risk prediction tool. This was then compared with the criteria used in the wider Targeted Lung Health Checks programme. The prediction tool outperformed by detecting 822 cases compared to 581 by the lung check programme currently in operation.

**Conclusion:**

We have demonstrated the useful application of Machine Learning in developing a risk score for lung cancer and discuss its clinical applicability.

## Introduction

Lung cancer is one of the major causes of death worldwide [1, 2]. In the UK, 48,500 new lung cancer cases are detected every year of which 34,800 die, accounting for 21% of all cancer deaths during 2017–2019 [3]. An estimated 86% of lung cancer deaths in the UK are caused by tobacco smoking [4]. Furthermore, there is an association with prolonged environmental exposure to air pollutants such as sulphur dioxide, nitrogen oxides, nitrogen dioxides, or arsenic. Hence, nations with greater pollution levels are likely to have higher incidences of lung cancer [5]. Until the advent of the Targeted Lung Health Check (TLHC) pilots, it was only when a person started to exhibit the symptoms of lung cancer, that a diagnosis of the disease could be made. Some of these symptoms could include coughing, shortness of breath, unexplained weight loss, wheezing, haemoptysis, chest discomfort, exhaustion and decreased appetite [6].

Lung cancer outcomes have improved only marginally over the last 40 years and remain poor in comparison to most other cancers—just 17.7% of women and 12.9% of men in the UK survive after diagnosis for 5 years or longer [7]. The lack of overt or specific symptoms in the early stages of lung cancer often leads to late presentations, resulting in delayed diagnosis and treatment [8]. However, early detection and diagnosis, followed by effective treatment, improves survival for nearly all cancer types. According to Cancer Research UK [9] “around 6 in 10 people with lung cancer survive their disease for 5 years or more, if diagnosed at the earliest stage. This falls to <1 in 10 people when lung cancer is diagnosed at the most advanced stage.”

When diagnosed early, more treatment options are available for lung cancer, including surgical resections. If operable, primary treatment costs are largely attributable to surgical removal procedures. However, as the disease advances to Stages 3 and 4, the expenses associated with surgical interventions tend to decrease, whilst the costs related to systemic therapies escalate significantly. This shift in treatment modalities is primarily due to the diminished feasibility of surgical removal as the cancer becomes more widespread. Instead, systemic therapies become more pivotal at advanced stages, aiming to control tumour growth, alleviate symptoms and potentially prolong survival. Consequently, timely identification and detection of lung cancer can significantly alleviate the financial burden on the state, the insurer, patients, and their families. This includes mitigating the expenses associated with advanced-stage treatments, extended hospital stays, intensive therapies, and palliative care services [10].

In the quest for earlier diagnosis of lung cancer, in June 2023, the UK government announced plans for a new national targeted lung cancer screening programme, based on learning from existing Targeted Lung Health Check (TLHC) pilot sites. The programme, which is supported by a recommendation from the UK National Screening Committee, will invite patients aged between 55 and 74 who are current or former smokers for a lung health check, which may include a low-dose CT scan. In areas where the TLHC programme has been operating, early data suggests that approximately 76% of lung cancers are diagnosed at stages 1 and 2, which is a substantial improvement compared with usual pathways of care [11].

Artificial Intelligence (AI) is a new and rapidly evolving field where computers are taught to think like humans. Due to its enhanced accuracy, precision, and decision-support capabilities, AI has begun to be implemented in modern medicine. It is being used in two ways namely, physical and virtual. Physical applications of AI include robots that are automated to perform tasks such as caring for the elderly and others that assist in surgeries. Machine learning (ML) is a subfield of AI that deals with the virtual aspect. ML models can be trained to detect or predict occurrences of a health condition [12]. AI is suitable in the medical field as it has no concept of fatigue unlike doctors and therefore can process large number of images and data at any given time [13]. This requires a good prediction model to be designed which involves acquiring a large dataset for training the model. The bigger and more diverse the dataset is, better the results that can be expected from it [14]. However, researchers need to be aware that quality, curation, and expert annotation are vitally important while considering what data to include.

With the help of AI, we can make accurate assessments of one’s risk of lung cancer. The detection or prediction of lung cancer serves as a prime illustration where the utilisation of AI is indispensable. This is due to the fact that lung cancer is a highly time-sensitive condition and early diagnosis can be difference between life and death. Risk factors associated with lifestyle choices can be used to provide profiles of potential risks. The objective of any risk prediction tool, such as the one described in this paper, is to identify a small fraction of the population in which a large proportion of the disease cases will occur [15].

The National Screening Committee has recommended population screening for lung cancer as targeted lung cancer screening with low-dose Computerised Tomography is cost-effective at a threshold of £20,000 per QALY [16, 17]. Current attempts to improve early lung cancer diagnosis involve diagnostically evaluating large volumes of individuals with less than 1% of successful case identification [18, 19]. The population of England is estimated to increase by 6% over the next decade [20]. Furthermore, there has been a 19% increase in the prevalence of cancer in England over the last decade and published figures on the number of people waiting for a diagnosis or treatment for cancer have shown the huge challenge facing NHS cancer services, with tens of thousands of people waiting too long for diagnosis or vital treatment, especially since the start of the pandemic of COVID-19 [21]. Hence, the NHS cannot afford to provide existing healthcare in the same way in the future and will not have a sufficient workforce to deliver this. This challenge is not just isolated to the UK but is a common issue worldwide.

Our study aims to address the challenge of delayed diagnosis of lung cancer by exploiting the processing power of AI. We developed a model for providing risk-based predictions of lung cancer based on an individual’s lifestyle choices, family history and other clinical data. We had access to a large dataset consisting of 1.25 million adult residents across the Kent and Medway region called the Kent Integrated Dataset (KID) [22]. We harnessed the capabilities of ML to train the model in making risk predictions by extracting patterns from data records of residents who had been diagnosed and treated for lung cancer. Our objective was to find the best performing model among a group of ML models that gave accurate predictions of the risk of lung cancer.

## Methods

### The County of Kent

Kent County Council covers the largest population footprint of any other council in England with a population of 1.6 million [23]. It has an exceptional spread of affluence and extreme poverty. Before COVID, a life expectancy gap of almost 20 years already existed between the least and most deprived wards [24, 25].

### Dataset description

Data for this study was taken from the KID [22], which contains a vast array of pseudonymised integrated health and care data. The data for KID are derived from various sources. Nearly 40% of the data is from secondary care, over ¼ from primary care and the rest are from a range of sources including community and mental health trust providers and other publicly available data at a spatial level. The KID is overseen by a steering group known as the Kent & Medway Shared Health and Care Analytics Board (SHcAB) that includes representatives of Kent County Council, local health commissioners and information governance leads. The SHcAB considers issues such as information governance, development of the dataset and applications for use of the data. The Kent and Medway data warehouse team provides day-to-day administration and project management. Access was granted to the first author by the SHcAB for the study duration through established due process. Patients can opt-out of contributing data to the KID by informing their GP surgery that they do not want their data to be shared with external organisations. It has to be appreciated that the data is not in the public domain and it is a pseudonymised person level data set for most of the variables. We established a project oversight group, supported by the Kent & Medway cancer alliance which included cancer clinicians, service managers, Public Health physicians, epidemiologists, and AI experts. Regular stakeholder engagement took place throughout the study involving patients and public representatives.

Data contained within the KID represented a 6-year longitudinal record of health and care data for residents for 2014–2019 which was 1,865,382. An initial exclusion for under 18s years was made (*n* = 599,866) which reduced the cohort to 1,265,516. We then removed a further 10,532 patients (0.8% of the total population), due to incomplete or missing records data (for example smoking status), which took the original cohort size down to 1,254,984. We used a set of pre-determined criteria to exclude the records with missing data. Given that recording of ethnicity is poor across the NHS, we did not use it as an exclusion criteria. We excluded records where data on one or more key variables relevant to our analysis were missing. These are: Pseudonymised Unique Patient ID, Smoking Status of the individual, GP Practice of Registration, Age, Gender, and valid Postcode. The final dataset contained a total of 1,254,984 patients, of which 6053 were diagnosed with primary lung cancer during this period and these were included within the scope of this investigation. The final dataset used in the analysis had no missing data on smoking status. The cohort selection (lung cancer cohort) was only made up of patients with primary malignant lung cancers, excluding benign tumours and metastases from other types of cancer. To ensure comprehensive capture of all patients meeting the criteria, we assessed both primary and secondary healthcare records using relevant SNOMED or ICD-10 codes, respectively. Patients with Lung Cancer included all confirmed diagnoses regardless of diagnosis of care setting, staging at the time of diagnosis, disease progression or onward treatment options and outcomes. Core dimensions of data used within this study are shown below:

- Patient Demographics
- Primary Care (Events, Consultations, Long term condition registers, Medications, Deaths)
- Secondary Care (A&E, Inpatient Spells and Outpatients, Critical Care Bed Days)
- Mental Health (Inpatient and Outpatient History)
- Community Care (Contacts, Appointments, Minor Injuries Units and Walk In Centers)
- Wider Health Determinants including Housing, Education, Employment, and Income.
- Environmental Datasets—Pollution, Radon ground levels

We did not have information on all the above variables at an individual patient level. We had individual patient-level data on patient demographics, primary, secondary care, mental health and community clinical care activities. For the wider determinants of health including environmental factors, we applied spatial level data at the Lower layer Super Out Put area, a small geographical area in the UK with an average of 650 households to the patient level datasets.

### Data access

All NHS organisations including general practices across Kent & Medway had entered into Joint data Controller arrangements, which includes a common process for safe, secure and lawful access to their data in the KID for population health analytics including work such as ours. This process is administered by a system wide oversight group representing the organisations, called the Kent & Medway Shared Health & Care Analytics Board. Patient-level consent would not apply in this context as the dataset is historical and fully pseudonymised and deidentified. Because of the above arrangements, access to the data in KID, its analysis and sharing of the findings, no ethical approval was required as per existing arrangements.

### Data pre-processing

The dataset contained missing values mainly in the attribute named ‘ethnicity’ as shown in Table 1, despite a lot of work to try and capture ethnicity coding from various sources. We, therefore, excluded this from the model as we felt that it was not appropriate to try and use average value or synthetic data derivative, which is common practice. Other dataset attributes had no missing or outlier values from features, so no further transformations were made on the remainder of the datasets.

**Table 1:** Baseline characteristics of cohort groups.

Features	Lung Cancer Cohort (*n* = 6053)	Non-lung cancer cohort (*n* = 1,248,931)	Whole cohort (*n* = 1,254,984)
Age (Years)			
18–25	103 (1.8%)	150,304 (12%)	150,407 (12%)
26–44	642 (10.6%)	378,802 (30.5%)	379,444 (30.3%)
45–59	1241 (20.5%)	324,581 (26%)	325,822 (26%)
60+	4067 (67.1%)	395,244 (31.5%)	399,311 (31.7%)
Gastroenterological Disorders			
Yes	537 (8.9%)	55,814 (4.5%)	56,351 (4.5%)
No	5516 (91.1%)	1,193,117 (95.5%)	1,198,633 (95.5%)
Race (%)			
White - British	2281 (37.8%)	411,159 (33.1%)	413,440 (33.1%)
White - Any other White background	62 (1%)	17,838 (1.4%)	17,900 (1.4%)
White – Irish	18 (0.4%)	1626 (0.1%)	1644 (0.1%)
Black or Black British - Caribbean	2 (0%)	813 (0.1%)	815 (0.1%)
Black or Black British - African	11 (0.2%)	3667 (0.3%)	3678 (0.3%)
Black or Black British - Any other Black background	6 (0.1%)	2002 (0.2%)	2008 (0.2%)
Asian or Asian British - Bangladeshi	1 (0%)	832 (0.1%)	833 (0.1%)
Asian or Asian British - Pakistani	2 (0%)	746 (0.1%)	748 (0.1%)
Asian or Asian British - Indian	15 (0.2%)	5640 (0.5%)	5655 (0.5%)
Asian or Asian British - Any other Asian background	12 (0.2%)	3762 (0.3%)	3774 (0.3%)
Mixed - White and Black African	0 (0%)	485 (0%)	485 (0%)
Mixed - White and Black Caribbean	0 (0%)	591 (0%)	591 (0%)
Mixed - White and Asian	1 (0%)	762 (0.1%)	763 (0.1%)
Mixed - Any other mixed background	2 (0%)	1868 (0.1%)	1870 (0.1%)
Other Ethnic Groups - Chinese	3 (0%)	928 (0.1%)	931 (0.1%)
Other Ethnic Groups - Any other ethnic group	19 (0.3%)	5344 (0.4%)	5363 (0.4%)
Not stated	680 (11.3%)	98,172 (7.9%)	98,852 (7.9%)
Not known	2938 (48.5%)	686,643 (55.2%)	689,581 (55.2%)
Smoking Status (%)			
Never Smoked	968 (16%)	392,289 (31.4%)	393,257 (31.4%)
Passive Smoker/Ex-Trivial Smoker (<1 a day)	1110 (18.3%)	275,656 (22.1%)	276,766 (22.1%)
Trivial Smoker (<1 a day)/Ex-Light Smoker (1–9 a day)	691 (11.4%)	141,641 (11.3%)	142,332 (11.3%)
Light Smoker (1–9 a day) Ex-Moderate Smoker (10–19 a day)	1117 (18.5%)	22,2730 (17.8%)	223,847 (17.8%)
Moderate Smoker (10–19 a day)/Ex-Heavy Smoker (20+ a day)	1745 (28.8%)	186,827 (15%)	188,572 (15%)
Heavy Smoker (20+ a day)	422 (7%)	29788 (2.4%)	30210 (2.4%)
Care Home (%)			
Care Home	51 (0.8%)	6946 (0.6%)	6997 (0.6%)
Not in a Care Home	6002 (99.2%)	1,241,985 (99.4%)	1,247,987 (99.4%)
Deprivation (Decile)			
1 - Most Deprived	390 (6.4%)	75,207 (6%)	75,597 (6%)
2	546 (9%)	107,944 (8.6%)	108,490 (8.6%)
3	525 (8.7%)	105,137 (8.4%)	105,662 (8.4%)
4	665 (11%)	126,404 (10.1%)	127,069 (10.1%)
5	812 (13.4%)	158,157 (12.7%)	158,969 (12.7%)
6	683 (11.3%)	134,822 (10.8%)	135,505 (10.8%)
7	776 (12.8%)	165,452 (13.2%)	166,228 (13.2%)
8	655 (10.8%)	127,382 (10.2%)	128,037 (10.2%)
9	516 (8.5%)	117,639 (9.4%)	118,155 (9.4%)
10 - Least Deprived	451 (7.5%)	119,876 (9.6%)	120,327 (9.6%)
Unknown	34 (0.6%)	10,911 (0.9%)	10,945 (0.9%)
Population Segmentation Clusters (ACORN)			
Affluent Achievers	1490 (24.6%)	297,983 (24%)	299,473 (24%)
Comfortable Communities	1905 (31.5%)	381,269 (31%)	383,174 (31%)
Financially Stretched	1364 (22.5%)	256,201 (21%)	257,565 (21%)
Not Private Households	45 (0.7%)	8563 (1%)	8608 (1%)
Rising Prosperity	233 (3.8%)	68,672 (6%)	68,905 (6%)
Urban Adversity	707 (11.7%)	16,4400 (13%)	16,5107 (13%)
Undefined	309 (5.2%)	71,843 (6%)	72,152 (6%)
COPD			
Yes	1579 (26.1%)	185,039 (14.8%)	186,618 (14.8%)
No	4306 (71.1%)	1,020,885 (81.8%)	1,025,191 (81.8%)
Family History	168 (2.8%)	43,007 (3.4%)	43,175 (3.4%)
Hypertension			
Yes	1855 (30.6%)	210,788 (16.9%)	212,643 (16.9%)
No	3900 (64.4%)	952,750 (76.3%)	956,650 (76.3%)
Family History	298 (5%)	85,393 (6.8%)	85,691 (6.8%)
Diabetes			
Yes	2003 (33.1%)	278,378 (22.2%)	280,381 (22.2%)
No	3953 (65.3%)	943,729 (75.6%)	947,682 (75.6%)
Family History	97 (1.6%)	26,824 (2.2%)	26,921 (2.2%)
Tuberculousis			
Yes	75 (1.2%)	4823 (0.4%)	4898 (0.4%)
No	5961 (98.5%)	1,242,324 (99.5%)	1,248,285 (99.5%)
Family History	17 (0.3%)	1784 (0.1%)	1801 (0.1%)
Activity (%)			
Competitive Athlete	1 (0%)	267 (0%)	268 (0%)
Heavy (3+ days a week)	342 (5.7%)	90,414 (7.2%)	90,756 (7.2%)
Intermediate (2 Days a week)	4092 (67.6%)	905,749 (72.5%)	909,841 (72.5%)
Light (1 day a week)	912 (15%)	143,704 (11.6%)	144,616 (11.6%)
Rarely ( < 1 day a week)	652 (10.8%)	103,798 (8.3%)	104,450 (8.3%)
Exercise Impossible	54 (0.9%)	4999 (0.4%)	5053 (0.4%)
Other Cancers			
Yes (excludes lung cancer)	1281 (21.2%)	116,998 (9.4%)	118,279 (9.4%)
No	4354 (71.9%)	1,058,046 (84.7%)	1062,400 (84.7%)
Family History	418 (6.9%)	73,887 (5.9%)	74,305 (5.9%)
Cardiac Disorders			
Yes	2093 (34.6%)	207,638 (16.6%)	20,9731 (16.7%)
No	3436 (56.8%)	991,171 (79.4%)	994,607 (79.3%)
Family History	524 (8.7%)	50,122 (4%)	50,646 (4%)
Respiratory Disorders			
Yes	3845 (63.5%)	670,351 (53.7%)	674,196 (53.7%)
No	2122 (35.1%)	559,762 (44.8%)	561,884 (44.8%)
Family History	86 (1.4%)	18,818 (1.5%)	18,904 (1.5%)
Male			
Yes	2916 (48.2%)	607,295 (48.6%)	610,211 (48.6%)
No	3137 (51.8%)	641,627 (51.4%)	644,764 (51.4%)
Unknown	0 (0%)	9 (0%)	9 (0%)
Female			
Yes	3137 (51.8%)	641,627 (51.4%)	644,764 (51.4%)
No	2916 (48.2%)	607,295 (48.6%)	610,211 (48.6%)
Family History	0 (0%)	9 (0%)	9 (0%)

**Fig. 1:**
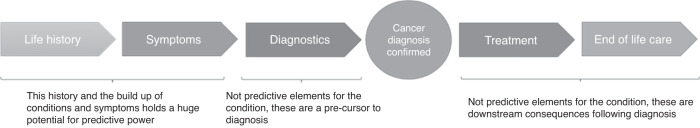
Pathways leading up to and beyond a Lung Cancer Diagnosis for patients. The model uses only life history and symptoms as predictive elements for a lung cancer diagnosis. Diagnostic elements, treatment and end of life care features were omitted.

The data attributes are grouped into life history, symptoms, diagnostics, treatment, and end-of-life care based on the stage at which the data are collected, as depicted in Fig. 1. To prepare the model for predicting patients’ risk ratios, we extracted only the essential attributes from the dataset. These columns were selected based on their potential to provide valuable predictive information. We specifically focused on data concerning the pathways leading to the diagnosis of lung cancer as it held valuable insights regarding the associated causes and symptoms. Attributes related to cancer diagnosis or data related to 2-week wait urgent referrals, appointments to see an oncologist, Chest X-Rays and Low Dose Computerised Tomography scans for confirming diagnosis, treatment options such as chemotherapy and radiotherapy and mortality were omitted. These attributes were excluded from the dataset because they were deemed as non-predictive elements that did not offer significant insights into the associated risks of a positive diagnosis of lung cancer. We excluded the above diagnostics and treatment elements up to 12 months before the date of diagnosis.

Relative risks (RR) were calculated for all the variables and were used to determine the important attributes and for categorisation. RR is the ratio of the incidence of an event occurring (Lung Cancer) with an exposure (e.g., smoking) versus the incidence of the same event occurring without the exposure. For example, the relative risk of developing lung cancer in smokers (the exposed group) versus non-smokers (non-exposed group) would be the probability of developing lung cancer for smokers divided by the probability of developing lung cancer for non-smokers. All characteristics of the individual datasets such as medications, events, tests, demographic qualities or wider determinant of health factors were tested, and risk-scored using this methodology. To reduce the number of categories we collapsed these into meaningful groupings, and these were informed by the higher relative risk of related variables. For instance, for respiratory disorders such as COPD and Asthma each of which have numerous diagnosis codes, these were built up into simple three-state options; Yes, No or Has Familial History. Other features, such as smoking history and activity with high dimensionality were ranked into similar groups by creating scores.

### Model development

We used feature encoding to reduce the number of states and to simplify the complexity of model development and enhance performance. One-hot encoding and standard scaling was used for the feature encoding [26]. Given the need to develop a scalar response to risk scoring in order to aid prioritisation of patients at greatest risk of developing lung cancer within a screening pool, logistical and other categorical models were ruled out. Traditional linear regression was selected as an initial candidate model to detect lung cancers early and thereby improving outcomes over and above the current screening protocol for lung cancer in the UK.

**Fig. 2:**
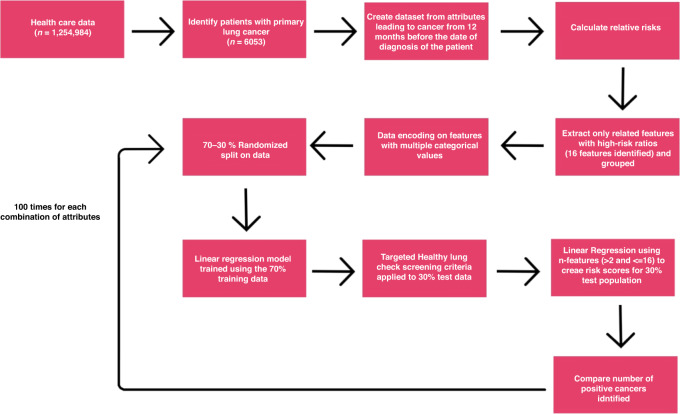
Steps involved from the beginning to the end of the study process. This spans from extracting relevant data from the KID to comparing the number of lung cancer cases detected using the most successful model and the criteria used in the TLHC programme.

Using a combination of methods namely informed by the data, proposals from clinical experts and published literature [27, 28], 16 attributes were identified. We took our entire population data for n attributes, which could be anywhere between 2 and 16, and split this into 70% training and 30% validation datasets [29]. We then used the 70% dataset to build a linear regression model on these n attributes. We developed a loop within Python [30] to identify all the possible combinations of these 16 attributes in their ability to detect lung cancer. We applied this model for n attributes to the 30% test population to achieve an output which is number of lung cancer cases detected. This was repeated one hundred times (Fig. 2) in order to create multiple outputs that could be averaged to test the models’ repeatability and for onward evaluation. We then employed boot strapping [31] to test the general ability of the model to work across randomised populations. In each run, both the 70% training set and the 30% validation set were again randomised to eliminate any potential biases or chance influences. This randomisation also aimed to provide comprehensive average performance statistics for all models. In each model run the TLHC eligibility criteria were applied, and the number of cancers counted. This was compared to the highest risk scored patients identified by the prediction model, keeping both the screening cohort sizes equal.

#### Model evaluation

The output of this model is not binary/logistical (with or without cancer) but a continuum of risk of developing the cancer. As we have stated within the dataset description section, the dataset we used also did not contain person-level information on all the variables included in the model. Hence, the traditional parameters to express the validity of a screening test such as sensitivity, specificity, positive predictive value, negative predictive value, area under the curve and likelihood ratios are not applicable. Instead, we rationalised that if the model is working most efficiently, we should be able to demonstrate more lung cancer cases being found within a screening pool in the population compared to that of the current screening pilots ongoing in England. In order to baseline our evaluation, therefore, we compared the output of the model against the current screening population for the TLHC [32] programme. Patients meeting the following three criteria will be invited for screening:

- are over 55 but younger than 75 years old
- are registered with an GP in the area the scheme is operating
- have ever smoked, and this is recorded with the GP.

This number of cases found from the TLHC programme was then compared with the number of cases identified using the linear regression model using the top-performing combination of attributes.

## Results

Selected characteristics of cohorts included in the study are shown in Table 1.

Relative risks for the attributes included in the model are presented in Table 2.

**Table 2:** Relative risks for the attributes and various levels of exposures included in the model.

Model Attribute	Attribute Category (Exposure)	With Lung cancer	Without Lung Cancer	Total	Incidence Among Exposed	Incidence Among Unexposed	Relative Risk	95% Confidence Interval	
								Lower	Upper
Activity	Competitive Athlete	1	267	268	0.0037313	0.0048234	0.77	0.11	5.47
	Heavy (3+ days a week)	342	90,414	90,756	0.0037683	0.0049054	0.77	0.69	0.86
	Intermediate (2 Days a week)	4092	905,749	909,841	0.0044975	0.0056817	0.79	0.75	0.84
	Light (1 day a week)	912	143,704	144,616	0.0063064	0.0046300	1.36	1.27	1.46
	Rarely (<1 day a week)	652	103,798	104,450	0.0062422	0.0046943	1.33	1.23	1.44
	Exercise Impossible	54	4999	5053	0.0106867	0.0047995	2.23	1.71	2.91
Male	Yes	2916	607,295	610,211	0.0047787	0.0048653	0.98	0.93	1.03
	No	3137	641,627	644,764	0.0048653	0.0047786	1.02	0.97	1.07
	Unknown	0	9	9	0.0000000	0.0048232	0.00	0.00	0.00
Female	Yes	3137	641,627	644,764	0.0048653	0.0047786	1.02	0.97	1.07
	No	2916	607,295	610,211	0.0048653	0.0047786	1.02	0.97	1.07
	Unknown	0	9	9	0.0000000	0.0048232	0.00	0.00	0.00
Age	18–25	103	150,304	150,407	0.0006848	0.0053867	0.13	0.10	0.15
	26–44	642	378,802	379,444	0.0016919	0.0061802	0.27	0.25	0.30
	45–59	1241	324,581	325,822	0.0038088	0.0051789	0.74	0.69	0.78
	60+	4067	395,244	399,311	0.0101850	0.0023210	4.39	4.16	4.63
COPD	Yes	1579	185,039	186,618	0.0084611	0.0041877	2.02	1.91	2.14
	No	4306	1,020,885	1,025,191	0.0042002	0.0076025	0.55	0.52	0.58
	Family History	168	43,007	43,175	0.0038911	0.0048564	0.80	0.69	0.93
Hypertension	No	3900	952,750	956,650	0.0040767	0.0072167	0.56	0.54	0.60
	Family History	298	85,393	85,691	0.0034776	0.0049218	0.71	0.63	0.79
	Has	1855	210,788	212,643	0.0087235	0.0040275	2.17	2.05	2.29
Diabetes	Yes	2003	278,378	280,381	0.0071439	0.0041555	1.72	1.63	1.81
	No	3953	943,729	947,682	0.0041712	0.0068337	0.61	0.58	0.64
	Family History	97	26,824	26,921	0.0036031	0.0048499	0.74	0.61	0.91
Cardiac Disorders	Yes	2093	207,638	209,731	0.0099794	0.0037886	2.63	2.50	2.78
	No	3436	991,171	994,607	0.0034546	0.0100508	0.34	0.33	0.36
	Family History	524	50,122	50,646	0.0103463	0.0045909	2.25	2.06	2.46
Respiratory Disorders	Yes	3845	670,351	674,196	0.0057031	0.0038017	1.50	1.42	1.58
	No	2122	559,762	561,884	0.0037766	0.0056716	0.67	0.63	0.70
	Family History	86	18,818	18,904	0.0045493	0.0048274	0.94	0.76	1.17
Gastroenterological Disorders	Yes	537	55,814	56,351	0.0095296	0.0046019	2.07	1.90	2.26
	No	5516	1,193,117	1,198,633	0.0046019	0.0095296	0.48	0.44	0.53
Other Cancers	Yes (excludes Lung Cancer)	1281	116,998	118,279	0.0108303	0.0041981	2.58	2.43	2.74
	No	4354	1,058,046	1,062,400	0.0040983	0.0088221	0.46	0.44	0.49
	Family History	418	73,887	74,305	0.0056255	0.0047727	1.18	1.07	1.30
Tuberculosis	Yes	75	4823	4898	0.0153124	0.0047821	3.20	2.55	4.01
	No	5961	1,242,324	1,248,285	0.0047754	0.0137334	0.35	0.28	0.43
	Family History	17	1784	1801	0.0094392	0.0048165	1.96	1.22	3.15
Smoking Status	Never Smoked	968	392,289	393,257	0.0024615	0.0059009	0.42	0.39	0.45
	Passive Smoker/Ex-Trivial Smoker (<1 a day)	1110	275,656	276,766	0.0040106	0.0050531	0.79	0.74	0.85
	Trivial Smoker (<1 a day)/Ex-Light Smoker (1–9 a day)	691	141,641	142,332	0.0048548	0.0048191	1.01	0.93	1.09
	Light Smoker (1–9 a day)/Ex-Moderate Smoker (10–19 a day)	1117	222,730	223,847	0.0049900	0.0047869	1.04	0.98	1.11
	Moderate Smoker (10–19 a day)/Ex-Heavy Smoker (20+ a day)	1745	186,827	188,572	0.0092538	0.0040397	2.29	2.17	2.42
	Heavy Smoker (20+ a day)	422	29,788	30,210	0.0139689	0.0045976	3.04	2.75	3.35
Deprivation Deciles	1 - Most Deprived	390	75,207	75,597	0.0051589	0.0048016	1.07	0.97	1.19
	2	546	107,944	1,08,490	0.0050327	0.0048033	1.05	0.96	1.14
	3	525	105,137	1,05,662	0.0049687	0.0048098	1.03	0.94	1.13
	4	665	126,404	1,27,069	0.0052334	0.0047770	1.10	1.01	1.19
	5	812	158,157	1,58,969	0.0051079	0.0047819	1.07	0.99	1.15
	6	683	134,822	1,35,505	0.0050404	0.0047969	1.05	0.97	1.14
	7	776	165,452	1,66,228	0.0046683	0.0048468	0.96	0.89	1.04
	8	655	127,382	1,28,037	0.0051157	0.0047899	1.07	0.99	1.16
	9	516	117,639	1,18,155	0.0043671	0.0048706	0.90	0.82	0.98
	10 - Least Deprived	451	119,876	1,20,327	0.0037481	0.0049372	0.76	0.69	0.84
	Unknown	34	10,911	10,945	0.0031064	0.0048383	0.64	0.46	0.90
Population Segmentation Clusters (ACORN)	Affluent Achievers	1490	297,983	2,99,473	0.0049754	0.0047755	1.04	0.98	1.10
	Comfortable Communities	1905	381,269	3,83,174	0.0049716	0.0047579	1.04	0.99	1.10
	Financially Stretched	1364	256,201	2,57,565	0.0052958	0.0047011	1.13	1.06	1.20
	Not Private Households	45	8563	8608	0.0052277	0.0048204	1.08	0.81	1.45
	Rising Prosperity	233	68,672	68,905	0.0033815	0.0049069	0.69	0.60	0.79
	Urban Adversity	707	164,400	1,65,107	0.0042821	0.0049051	0.87	0.81	0.94
	Undefined	309	71,843	72,152	0.0042826	0.0048561	0.88	0.79	0.99
Care Home (%)	Yes	51	6946	6997	0.0072888	0.0048093	1.52	1.15	1.99
	No	6002	1,241,985	12,47,987	0.0048093	0.0072888	0.66	0.50	0.87

In the attribute concerning family history of cancer, lung cancer is also included. Many attributes were associated with an increased risk of lung cancer and others a lower risk. As expected, key attributes showing a higher risk included older age, lack of physical activity, COPD, hypertension, other cancers and family history of other cancers, TB and family history of TB and financial status. Attributes associated with lower risk include intense physical activity, younger age, never smokers and higher socioeconomic status. As the results are from univariate linear regression the effect of confounding is apparent. For example, hypertension is associated with age.

The top ten combinations of attributes were selected which showed the best results in identifying lung cancers, out of many thousands of combinations (Table 3). The selected combinations contained attributes numbering from 7 to 11. The top performing combination included the following attributes: age; activity score; smoking score; any respiratory illness; hypertension; cancer; and Tuberculosis.

**Table 3:** Attributes included in the best performing models and cancer cases detected.

Combination of Attributes	No. of attributes in the Model	Model Runs	Lung Cancer Cases detected	95% CI^a^	
				Lower	Upper
Age, Activity score, Smoking score, Any respiratory, HT, Cancer, TB	7	100	822	827	817
Age, Active score, Smoking score, Any respiratory, HT, Cancer, TB, Male, Female	9	100	821	826	816
Age, Active score, Smoking score, Gastro, HT, Cancer, Any respiratory, Cancer, Male, Female	10	100	820	825	815
Age, Active score, Smoking score, Gastric condition, HT, Cancer, Any respiratory, TB	8	100	819	824	814
Age, Active score, Smoking score, COPD, Gastric condition, HT, Cancer, Respiratory disease, TB, Male, Female	11	100	818	822	813
Age, Active score, Smoking score, COPD, Gastric condition, HT, Cancer, TB, Male, Female	10	100	817	822	812
Age, Active score, Smoking score, COPD, Endocrine and metabolic condition, Gastric condition, Cancer, TB, Male, Female	10	100	817	822	812
Age, Active score, Smoking score, COPD, Gastric condition, HT, Cancer, Respiratory disease, TB	9	100	817	821	812
Age, Active score, Smoking score, COPD, Endocrine and metabolic condition, Gastric condition, Cancer, TB	8	100	817	821	812
Age, Active score, Smoking score, COPD, Gastric condition, HT, Cancer, TB	8	100	816	821	812

^a^Confidence interval

We needed to test the performance of the 7-attribute combination henceforth referred to as the Kent & Medway risk prediction tool with the TLHC eligibility criteria. By applying these three criteria to the 30% test population we identified on average 56,663 people (screening cohort) who will be eligible under the TLHC criteria. Among these there were 581 lung cancer cases recorded. We then applied the Kent & Medway risk prediction tool to the same 30% test population, and this predicted a lung cancer risk score for every individual. From this list, we identified the top 56,663 people and within this population 822 lung cancer cases were recorded. This was on average a benefit of 41.4% over and above the contemporaneous approach.

### Discussion

Our study is an attempt to develop a lung cancer risk prediction tool to identify sections of the population at a higher risk of developing lung cancer. We utilised data both at person and spatial level including on social, demographic, lifestyle and clinical features and used the power of ML to achieve our objective. We initially identified 16 attributes that could predict the population at a higher risk of lung cancer. Our objective was to increase the power of cancer detection in a defined population as the current targeted TLHC eligibility criteria [32] are too broad and blunt. By running simultaneous models using boot strapping we were able to test numerous combinations of attributes running into tens of thousands of model runs which provided us with the best model with 7 attributes. We adopted a linear regression model which is different to others who have employed a suite of models [33, 34] in lung cancer prediction literature. This is because our objective was to identify a cohort of people at higher risk of lung cancer so that they can be targeted for screening. There is a linear association with many known attributes and risk of lung cancer. Furthermore, lung cancer risk score which is our main outcome of interest is a continuous variable and hence logistic regression is not applicable here. Use of ML has been proposed and adopted in reading computer tomography images [34]. However, in our study we used data points derived from routine linked administrative data sets which contained information on every patient irrespective of their clinical characteristics to predict their risk of lung cancer by exploiting the potential of ML. It may be surprising that the data on smoking status was almost complete although, this is not usually the case especially in Primary Care but shows continuous improvement [35]. The potential reasons for such high completeness in our study include the following: The KID being a linked dataset enabling smoking status to be captured from multiple points of care. Due to its specialist nature, a lot of efforts and resources have been spent to retrospectively ensure that the data is as complete as possible so that epidemiological research can be undertaken at the population level [22].

### Clinical utility of the work

The product of this work has immediate clinical implications and thus has the potential to improve patient care and resource utilization. As the model outperforms the standard wider TLHC eligibility criteria, this would help us to detect up to 40% more cancers. Currently, we are exploring how best to incorporate this as a screening and early diagnosis intervention. There are two options under consideration: provide a more comprehensive and refined screening model based on our risk tool compared to that of the THLC eligibility criteria; and the GP calculates the risk score for each patient during a consultation, similar to Framingham cardiac risk score [36] and use this for further action. Using the first option, we can further refine the risk group for screening there by increasing cancer detection and saving scarce cancer diagnostic and treatment resources. We intend to incorporate the tool into the management information system of the early cancer diagnosis team at the local hospital as a pilot and then to roll it out across a wider geographical area. The first author has already secured agreement in principle for this from the local cancer clinical and managerial leaders.

### Strengths

We used a place-based linked data set entirely produced by a local health system whose primary use was for commissioning intelligence and health care planning purposes. It has the power of painting the entire picture of the population as it contains information from general practice, community health services, mental health services and hospital services. Furthermore, it included integrated spatial-level information on key socioeconomic factors and the extent of deprivation. This makes it a powerful repository to develop any risk prediction tool compared to tools that only rely on electronic patient clinical records [37]. Our data is complete compared to Callender et al. [38] where there are large number of missing values. We generated relative risks at a very granular level of detail in order to develop our aggregated sixteen attributes. We established a powerful partnership of cancer clinicians, Public Health physicians, epidemiologists, ML experts and leaders from the cancer alliance who were involved throughout from the inception of the project to its completion. This helped us to incorporate varying perspectives. Key stakeholders’ views were constantly sought and acted upon during this work. These included regular meetings with the early diagnosis team, digital cancer alliance board, shared health and care analytics board and regional applied research consortium digital innovation group. Patients and the public are represented in most of these in order to ensure that there is support for this initiative.

#### Limitations

A few limitations of our study need to be acknowledged. All the seven variables included in the model had complete data although this does need to be treated with the following caution. For the activity score, we used the data at a population level i.e. lower layer super output area. This does not reflect the score for an individual-specific patient. Data on socio economic status was also only available at a spatial level. Although using data at geographical/spatial level gives us the advantage of complete data with no missing values, one needs to be cognisant of the limitations of this approach and the well-documented ecological fallacy [39]. Four of the variables in the final model were purely clinical conditions. These are: Any Respiratory Illness, Hypertension, Cancer, and Tuberculosis. It is extremely unlikely that such an important diagnosis will be left uncoded both in primary and secondary care. It is generally agreed that if such a clinical diagnosis does not appear on the patient record, the patient does not have the condition as it is not current practice to code that a patient does not have a condition. We recognise that this may not be universally true for all patients, but is unlikely to have a significant impact upon our longitudinal study results. Both for passive smoking and family history of cancer we assumed that if this information is not coded then the individual does not have that exposure although this may not be always accurate. As our analysis included over a million records any under/over assumption is likely to be random and will not have a major impact on the results. Ethnicity was not included in the model because the data was incomplete. In the future, we will ensure that ethnicity is included in further work. Data included in the study is only up to 2019.

We wish to acknowledge that we have not used traditional parameters to express the validity of a screening test as this approach is not applicable as explained in the model evaluation section. We have used a different approach to evaluate the model. It is the authors’ belief that the approach adopted in this study still adds useful information to the literature as this method has been seldom applied. This needs to be borne in mind when interpreting the findings and developing any policy approach based upon our findings. Due to changes in commissioning arrangements, the KID was rendered static and data were not updated after 2019. We do not anticipate any weakening of the power of the prediction tool due to non-inclusion of more recent data. This study was undertaken in Kent & Medway in the southeast of England. Hence the question of generalisability across the United Kingdom needs to be considered. In our view, it is unlikely that the population and the strength of association between the attributes and lung cancer are so different elsewhere that the results will not be applicable. However, this may not be true for an international comparison. Another important limitation worthy of note is that applying similar machine-learning approaches using other databases with different characteristics may result in a less sensitive outcome. Hence, before our approach is adopted this needs to be tested on a much larger patient population under different settings.

## Conclusion

In this paper, we have demonstrated the useful application of Machine Learning in developing a risk score for lung cancer using a large, place-based linked data set. We involved multidisciplinary stakeholders throughout this work, including patients and the public. Our risk prediction tool is superior to the eligibility criteria currently in use in the pilot sites for the TLHC Programme. This is a good example where local experts in fields as diverse as AI, ML, clinical oncology, Public Health and Epidemiology came together to produce an innovative solution to improve patient care and save scarce health care resources.

## Data Availability

The data are not publicly available as the KID contains pseudonymised person-level linked data. However, access to data can be requested via the SHcAB.
